# DTF-YOLOv11n: An Improved YOLOv11n Model for Apple Mosaic Disease Severity Detection

**DOI:** 10.3390/plants15213262

**Published:** 2026-10-22

**Authors:** Wenxu Chen, Mengting Wang, Xianlong Wang, Zehua Fan

**Affiliations:** 1 School of Network Security, Tarim University, Alar 843300, China; 2 Key Laboratory of Tarim Oasis Agriculture, Ministry of Education, Tarim University, Alar 843300, China; 3 School of Information Engineering, Changji University, Changji 831100, China; 4 School of Water Resources and Hydro-Electric Engineering, Xi’an University of Technology, Xi’an 710048, China

**Keywords:** apple mosaic disease, disease severity detection, object detection, YOLOv11n, multiscale feature fusion, deep learning

## Abstract

Apple mosaic disease can impair leaf photosynthetic capacity, reducing apple yield and fruit quality. Accurate detection of leaf disease severity is essential for disease monitoring and developing effective management strategies. However, subtle phenotypic differences between adjacent disease severity levels, particularly in local chlorosis, mosaic texture, and lesion boundaries, make accurate severity discrimination challenging. These fine-grained disease features may also be attenuated during feature propagation and conventional fixed upsampling, further hindering severity detection. To address these issues, we adopted YOLOv11n as the baseline model and integrated DySample, Triplet Attention, and FasterNet-PConv to develop DTF-YOLOv11n for apple mosaic disease severity detection. In the Neck feature-fusion pathway, DySample replaced conventional fixed upsampling operations, enabling the model to learn adaptive sampling locations and better preserve local disease information during upsampling. We introduced Triplet Attention after fusing P3, P4, and P5 features to enhance disease-characteristic representation across severity levels through cross-dimensional feature interactions. In addition, we incorporated FasterNet-PConv into the Backbone’s deep feature extraction stage to reduce redundant spatial computation while controlling model complexity. Experimental results show that DTF-YOLOv11n achieved mean average precision (mAP) values of 91.31% at an intersection over union (IoU) threshold of 0.50 (mAP50) and 86.73% averaged across IoU thresholds of 0.50–0.95 (mAP50-95), representing relative improvements of 2.73% and 3.66% over YOLOv11n, while reducing the number of parameters from 2.591 M to 2.471 M. Compared with multiple YOLO-series and non-YOLO detection models, DTF-YOLOv11n demonstrated strong overall detection performance. The proposed model improves the representation of apple mosaic disease features across severity levels while maintaining relatively low model complexity, providing a technical reference for automated detection and severity grading of apple mosaic disease.

## 1. Introduction

Apples are one of China’s major economic fruit crops, with cultivation area and production consistently ranking among the highest worldwide [1,2,3]. As large-scale apple production expands, diseases have become a major factor affecting yield and fruit quality. Therefore, rapid and accurate disease detection is essential for timely disease management [4,5,6]. Among these diseases, apple mosaic disease is a common viral foliar disease characterized primarily by leaf chlorosis and mosaic symptoms; severe infection can impair photosynthesis and adversely affect fruit yield and quality [7,8,9]. As disease severity increases, the phenotypic characteristics of mosaic patterns and chlorosis, as well as their spatial extent on the leaves, also change. Consequently, simply determining whether a leaf is diseased is insufficient to adequately characterize disease severity [10,11,12]. Accurate grading of apple mosaic disease severity can therefore support the development of appropriate disease management strategies [13,14,15].

At present, assessment of apple leaf disease severity still relies heavily on manual field surveys and empirical judgment. This process is labor-intensive and susceptible to observer subjectivity, making it difficult to meet the demands of continuous monitoring in large-scale orchards [16,17,18]. Although traditional machine-learning methods have been applied to plant disease recognition, they generally depend on handcrafted features such as color, texture, and morphology, and their feature representation capability is limited under varying illumination and background interference [19,20,21]. To improve the objectivity and quantitative assessment of disease severity, Zhang et al. developed a spectral index for Marssonina blotch on apple leaves. They effectively estimated the diseased-area proportion at the leaf scale, demonstrating the potential of spectral information for quantitative disease severity assessment [12]. With the development of deep learning, convolutional neural networks can automatically extract hierarchical features, providing new technical approaches for plant disease recognition and detection [22,23,24]. Sun et al. constructed the AppleDisease5 dataset and proposed the MEAN-SSD model for apple leaf disease detection, demonstrating the feasibility of deep-learning-based object detection for this task [1]. Compared with methods that perform only image-level classification, object detection can simultaneously localize leaf targets and identify their categories in complex scenes, providing a practical approach for automated detection of apple leaf disease severity [25,26,27].

Building on these advances, achieving an appropriate balance among detection accuracy, feature representation capability, and computational efficiency has become a key challenge in further developing automated apple leaf disease detection [28,29,30]. As a representative family of one-stage object detectors, YOLO (You Only Look Once) models offer advantages such as fast detection, flexible architectures, and end-to-end inference, and have therefore been widely applied to agricultural disease detection tasks [31,32,33]. In recent years, researchers have mainly improved the performance of YOLO-based models for apple leaf disease detection through multiscale feature fusion, attention mechanisms, and lightweight network design [34,35,36]. For example, Deng et al. proposed ALD-YOLO, which combines lightweight feature extraction with a multiscale attention mechanism to jointly improve detection accuracy and computational efficiency [37]. Li et al. developed YOLO-APLD, which enhances multiscale feature fusion while simplifying the network architecture, thereby improving apple leaf disease detection performance and reducing model complexity [9].

Beyond disease-category identification [38,39,40], disease-severity assessment has been studied through physiological measurement, lesion-based quantification, and feature-based grading. For apple mosaic disease, Liu et al. used hyperspectral images and leaf chlorophyll content to assess severity [41]. With RGB images, Pal and Kumar combined leaf segmentation with severity classification [42], while Feng et al. used semi-supervised segmentation to grade wheat Fusarium head blight [43]. More closely related to apple leaves, Liu et al. segmented leaves and lesions to grade frog-eye leaf spot in complex backgrounds [44], and Bi et al. combined segmentation with watershed processing to assess severity when leaves overlap [45]. Other studies have focused on discriminative symptom features: Yu et al. guided feature learning toward lesion regions [46], whereas Li and Li combined multiscale lesion features with adaptive feature transmission for kiwifruit severity recognition [47]. These approaches differ in imaging requirements, annotation demands, and prediction outputs, leaving the simultaneous localization and severity grading of apple mosaic leaves in orchard RGB images an important task. Despite these advances, adjacent severity grades of the same disease can share similar visual symptoms, making distinctions based on local chlorosis, mosaic texture, lesion boundaries, and symptom distribution particularly challenging [41,44,47]. In addition, variations in illumination, occlusion by branches and leaves, and differences in leaf orientation under natural orchard conditions further increase the difficulty of severity discrimination [30,48,49]. Therefore, reducing the loss of local disease details while enhancing feature representation across scales and severity levels, without substantially increasing model complexity, remains an important issue in automated apple mosaic disease severity detection [50,51,52].

To address these issues, we constructed a dataset comprising four severity levels of apple mosaic disease—Healthy, Light, Moderate, and Severe—and developed DTF-YOLOv11n using YOLOv11n as the baseline model. DySample was selected for content-adaptive reconstruction during upsampling, Triplet Attention for enhancing severity-relevant features after multiscale fusion, and FasterNet-PConv for reducing redundant spatial computation while controlling model complexity. Accordingly, these modules were placed at the Neck upsampling layers, after the P3/P4/P5 fused feature blocks, and within the Backbone’s P4/P5 stages, respectively. To evaluate the proposed model, we conducted comparative experiments with multiple object detection models and ablation experiments to systematically assess DTF-YOLOv11n’s overall detection performance, as well as the individual and combined contributions of the introduced modules. This study aims to improve automated detection of different severity levels of apple mosaic disease and provide an effective approach for severity grading and monitoring.

## 2. Results

### 2.1. Performance Comparison Between YOLOv11n and DTF-YOLOv11n

Under the same dataset split, input image size, and training settings, Table 1 presents the test results for YOLOv11n and DTF-YOLOv11n. DTF-YOLOv11n achieved precision (P), recall (R), mAP50, and mAP50-95 values of 91.40%, 86.41%, 91.31%, and 86.73%, respectively, corresponding to relative improvements of 5.42%, 0.81%, 2.73%, and 3.66% over YOLOv11n. The number of parameters decreased from 2.591 M to 2.471 M, while the model size decreased from 5.238 MB to 5.010 MB, representing reductions of 4.63% and 4.35%, respectively. Floating-point operations (FLOPs) decreased only slightly, from 6.444 G to 6.417 G, corresponding to a reduction of 0.42%, and therefore remained essentially unchanged overall.

**Figure 1:**
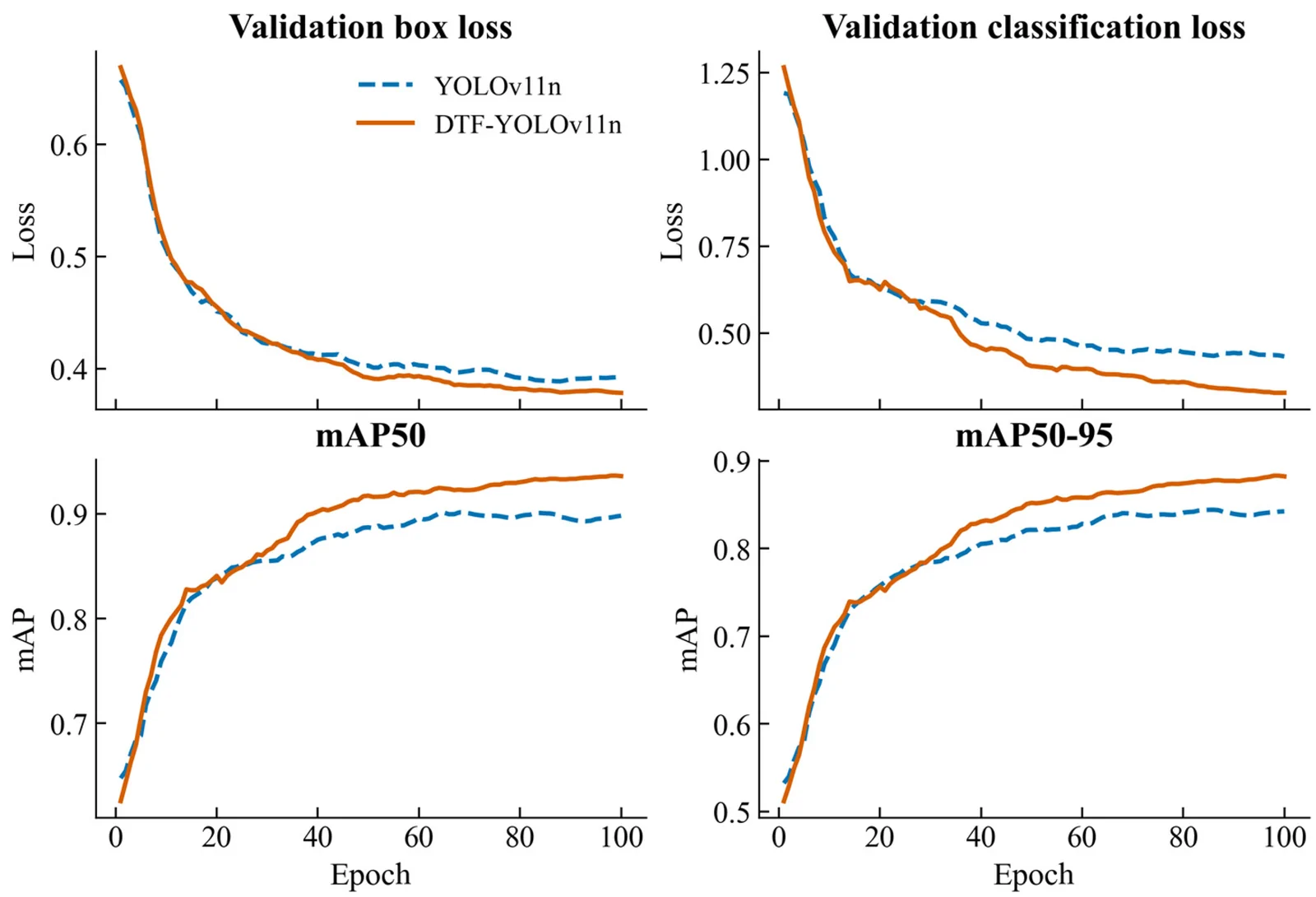
Validation loss and detection accuracy curves of YOLOv11n and DTF-YOLOv11n.

Figure 1 illustrates the changes in validation bounding-box loss, classification loss, mAP50, and mAP50-95 for YOLOv11n and DTF-YOLOv11n over 100 training epochs. Early in training, both models’ bounding-box and classification losses decreased rapidly, while mAP50 and mAP50-95 increased sharply. As training progressed, the magnitude of change in all metrics gradually diminished and eventually stabilized in later epochs, indicating that both models converged well. The overall trends in bounding-box loss were similar between the two models, although DTF-YOLOv11n remained slightly lower than YOLOv11n during the middle and later stages of training. In contrast, the classification loss difference was more pronounced, with DTF-YOLOv11n maintaining consistently lower values throughout the middle and later stages. In terms of detection accuracy, the mAP50 and mAP50-95 curves of the two models followed similar trends during the early epochs, after which the gap gradually widened. In the later stages of training, both metrics for DTF-YOLOv11n remained consistently higher than those of YOLOv11n, indicating that the improved model achieved better detection performance while maintaining stable convergence.

**Figure 2:**
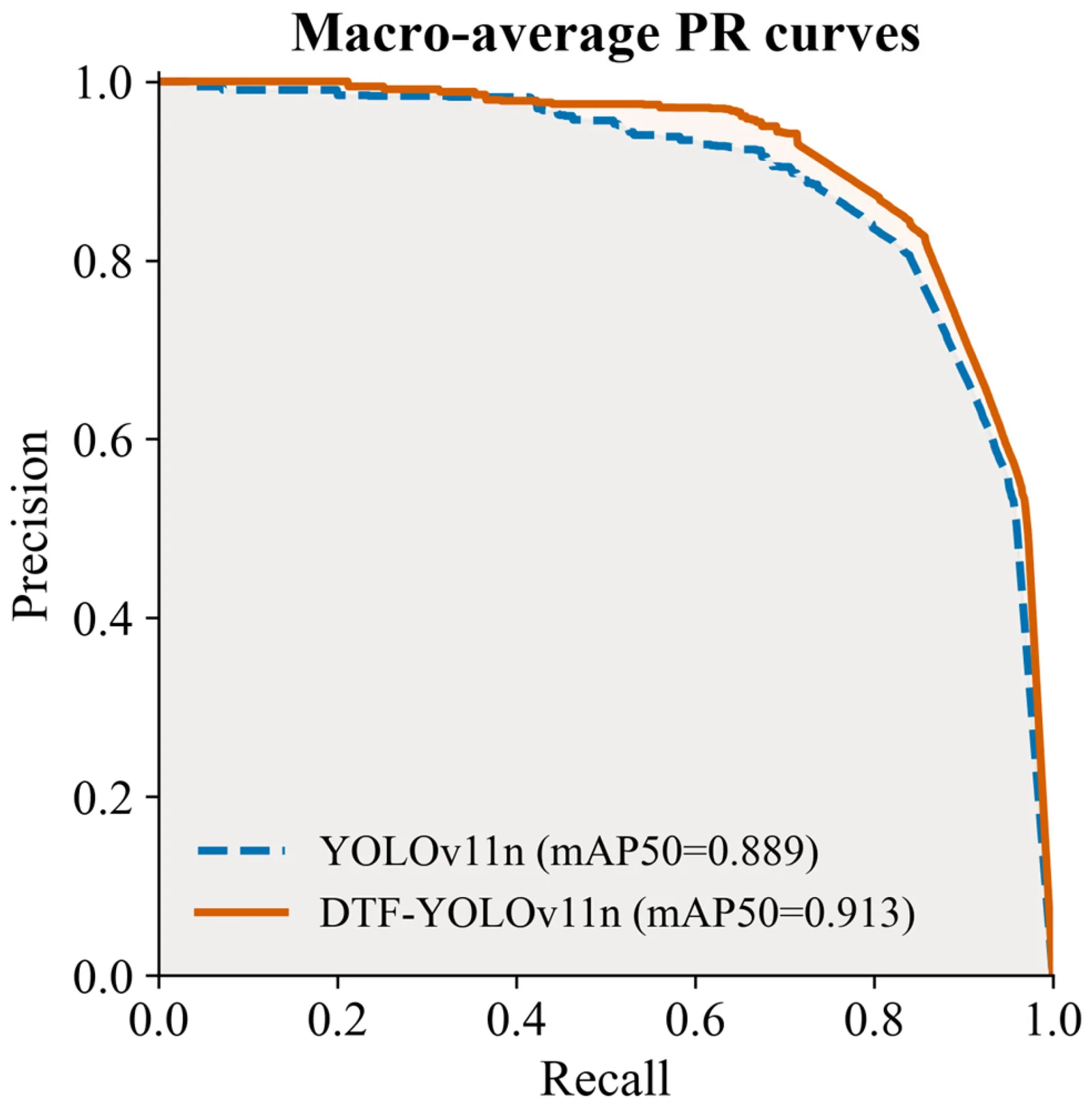
Macro-averaged PR curves of YOLOv11n and DTF-YOLOv11n on the test set.

Figure 2 presents the macro-averaged precision–recall (PR) curves of YOLOv11n and DTF-YOLOv11n on the test set. Both models maintained high P at relatively low R levels. As R increased, P gradually decreased, with a more pronounced decline in the high-R region. The two PR curves were similar in the low-R range but gradually diverged at medium-to-high R levels, with the DTF-YOLOv11n curve generally lying above that of YOLOv11n. This indicates that the improved model can maintain higher P as R increases. The mAP50 values of YOLOv11n and DTF-YOLOv11n were 88.88% and 91.31%, respectively, corresponding to a relative improvement of 2.73% for DTF-YOLOv11n. These results indicate that DTF-YOLOv11n achieves a better overall balance between P and R.

### 2.2. Performance Comparison of Different Object Detection Models

Table 2 presents the performance comparison of RetinaNet, SSDLite, and the YOLO-series models on the test set. All models used the same dataset split and evaluation metrics. The YOLO-series models were trained using consistent main training settings, whereas RetinaNet and SSDLite followed their respective training schemes. RetinaNet and SSDLite achieved mAP50 values of 74.58% and 75.23%, respectively, and mAP50-95 values of 64.96% and 57.16%, respectively, which were generally lower than those of the YOLO-series models. The mAP50 values of YOLOv5nu, YOLOv8n, YOLOv10n, YOLOv11n, and YOLOv12n ranged from 86.45% to 88.88%, while their mAP50-95 values ranged from 81.31% to 83.67%. Among these models, YOLOv11n achieved relatively high mAP50 and mAP50-95 values, whereas YOLOv12n achieved 86.69% and 82.23%, respectively, and did not surpass YOLOv11n. In comparison, DTF-YOLOv11n achieved P, R, mAP50, and mAP50-95 values of 91.40%, 86.41%, 91.31%, and 86.73%, respectively, all of which were the highest among the compared models. Meanwhile, its parameter count and model size were 2.471 M and 5.010 MB, respectively, both lower than those of the other YOLO-series models in the comparison. DTF-YOLOv11n recorded 106.2 frames per second (FPS), compared with 140.8 FPS for YOLOv11n, reflecting an accuracy–speed trade-off under the YOLO inference-stage timing definition.

**Figure 3:**
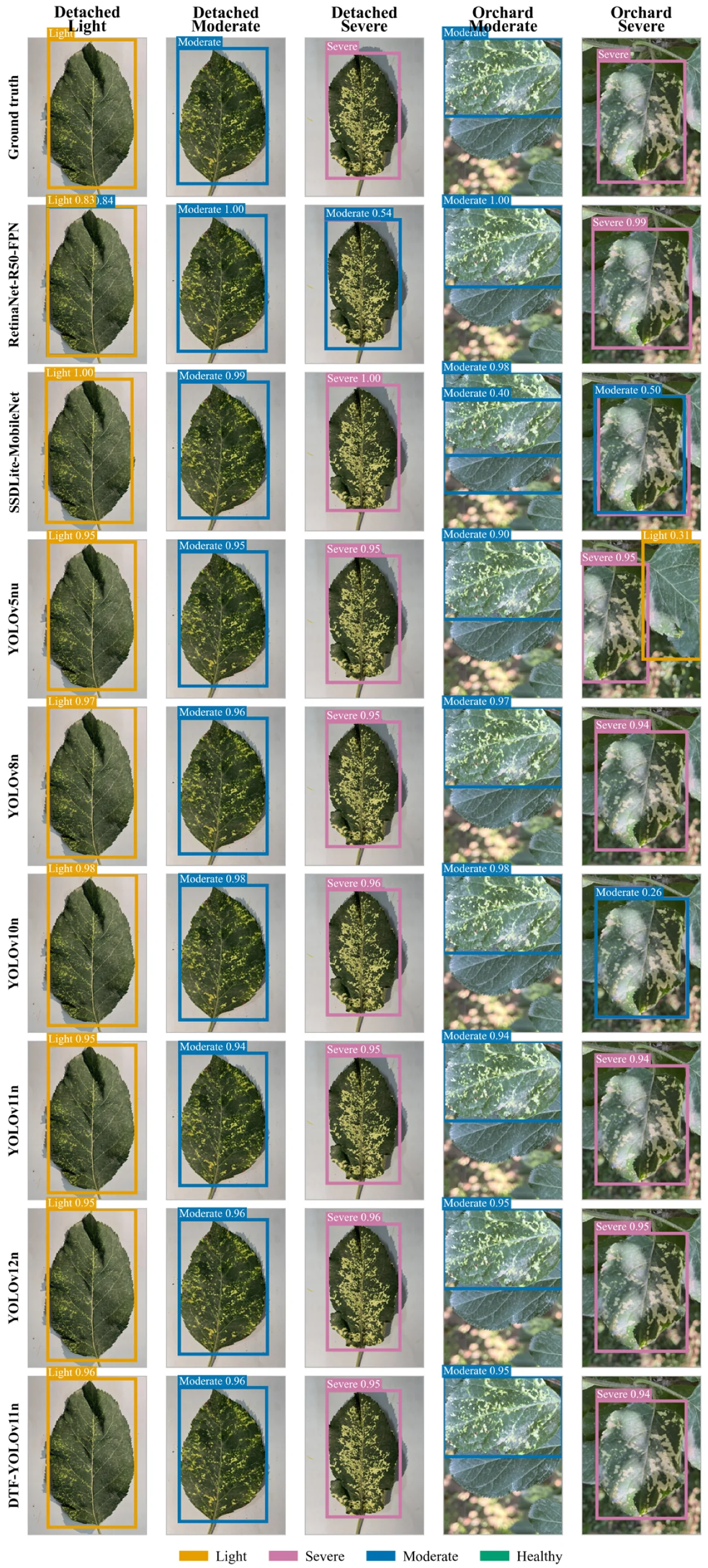
Comparison of detection results of different models on representative disease severity samples.

Figure 3 presents the detection results of different models on representative test samples from both detached-leaf and natural orchard scenes. Prediction results varied across models for the samples shown. For the detached light-severity sample, RetinaNet produced duplicate predictions of Light (0.83) and Moderate (0.84), and it classified the detached severe sample as Moderate with 0.54 confidence. SSDLite classified the severe sample from the orchard scene as Moderate with 0.50 confidence, while YOLOv10n also predicted Moderate with 0.26 confidence. In contrast, the predicted classes of DTF-YOLOv11n were consistent with the ground-truth labels for all five samples representing different severity levels and acquisition scenes, with corresponding confidence scores of 0.96, 0.96, 0.95, 0.95, and 0.94.

**Figure 4:**
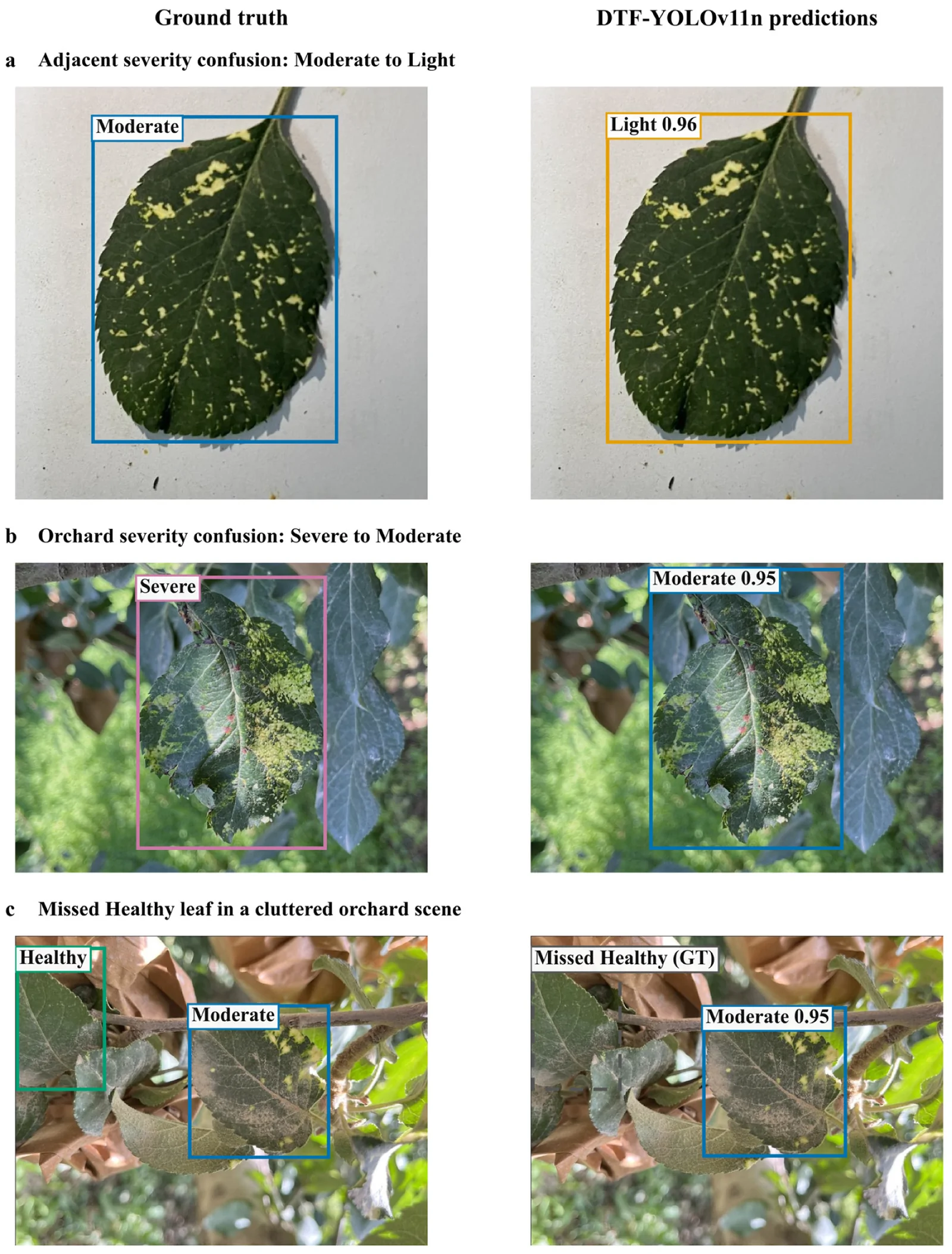
Representative failure cases of DTF-YOLOv11n on the test set. The left and right columns show ground-truth annotations and model predictions, respectively. (**a**) A Moderate leaf was predicted as Light. (**b**) A Severe leaf was predicted as Moderate in an orchard scene. (**c**) A partially visible Healthy leaf was missed, while the Moderate leaf in the same scene was detected. Numbers beside predicted classes indicate confidence scores. The dashed gray box marks the missed ground-truth target and is not a model prediction. Predictions are displayed at a confidence threshold of 0.25 and a non-maximum suppression IoU threshold of 0.50. Panel (**a**) was cropped identically in both columns for readability.

To examine the remaining detection errors, Figure 4 presents representative failure cases of DTF-YOLOv11n on the test set. These examples include confusion between adjacent severity categories and a missed Healthy leaf in a complex orchard scene.

### 2.3. Ablation Experiments of DTF-YOLOv11n

Table 3 presents the results of the eight ablation experiments. Compared with the baseline model, introducing DySample, Triplet Attention, and FasterNet-PConv individually improved both mAP50 and mAP50-95. Among the three single-module configurations, the DySample-only configuration achieved the highest mAP50 and mAP50-95 values. When FasterNet-PConv was introduced alone, parameters decreased from 2.591 M to 2.458 M, while FLOPs decreased from 6.444 G to 6.273 G.

All three two-module combinations achieved higher mAP50 values than the baseline model. Among them, the DySample and Triplet Attention combination achieved mAP50 and mAP50-95 values of 91.22% and 86.15%, respectively. Building on these results, the complete DTF-YOLOv11n further achieved mAP50 and mAP50-95 values of 91.31% and 86.73%, respectively, with 2.471 M parameters and 6.417 G FLOPs. The complete model achieved the highest mAP50 and mAP50-95 among the eight ablation configurations.

### 2.4. Detection Results for Different Severity Categories

Table 4 presents the average precision (AP) results averaged across IoU thresholds of 0.50–0.95 (AP50-95) for the different severity categories. DTF-YOLOv11n achieved AP50-95 values of 71.27%, 94.10%, 93.27%, and 88.27% for the Healthy, Light, Moderate, and Severe categories, respectively, representing relative improvements of 2.47%, 3.77%, 2.57%, and 5.70% over YOLOv11n. Among the four categories, the Severe category showed the largest relative improvement, the Light category achieved the highest AP50-95, and the Healthy category exhibited a relatively lower AP50-95.

**Figure 5:**
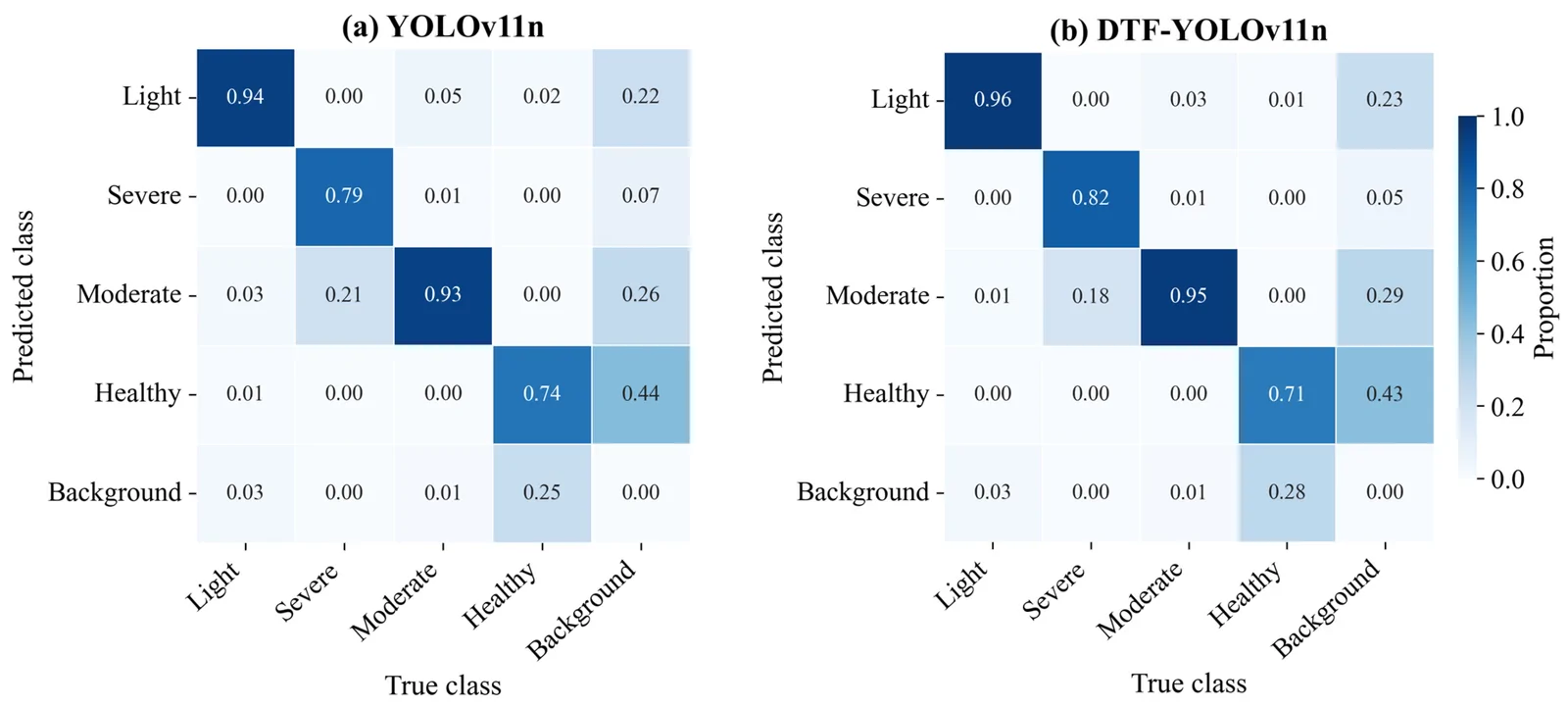
Normalized confusion matrices of YOLOv11n and DTF-YOLOv11n on the test set. (**a**) YOLOv11n. (**b**) DTF-YOLOv11n.

Figure 5 presents the normalized confusion matrices of YOLOv11n and DTF-YOLOv11n on the test set. For YOLOv11n, the diagonal proportions for the Light, Severe, Moderate, and Healthy categories were 0.94, 0.79, 0.93, and 0.74, respectively, whereas the corresponding values for DTF-YOLOv11n were 0.96, 0.82, 0.95, and 0.71. Specifically, the diagonal proportions for the Light, Severe, and Moderate categories increased from 0.94, 0.79, and 0.93 to 0.96, 0.82, and 0.95, respectively, while that of the Healthy category decreased from 0.74 to 0.71. Misclassification rates between adjacent severity categories also decreased: the proportion of Severe samples predicted as Moderate decreased from 0.21 to 0.18, that of Light samples predicted as Moderate decreased from 0.03 to 0.01, and that of Moderate samples predicted as Light decreased from 0.05 to 0.03. In addition, the proportion of Healthy samples predicted as Background increased from 0.25 to 0.28.

## 3. Discussion

### 3.1. Effects of Multi-Stage Feature Optimization on Overall Detection Performance

Compared with YOLOv11n, DTF-YOLOv11n achieved relative improvements of 2.73% and 3.66% in mAP50 and mAP50-95, respectively, for apple mosaic disease severity detection, with a more pronounced improvement in mAP50-95. This improvement is associated with the joint optimization of three stages: feature upsampling, fused feature enhancement, and deep feature extraction. DySample reconstructs features by learning sampling locations, thereby reducing the loss of spatial details during scale recovery compared with fixed upsampling. Triplet Attention enhances informative content in fused features through cross-dimensional feature interactions. Previous studies have shown that strengthening multiscale information propagation and feature representation in key regions can improve the extraction and localization of fine-grained disease features in plant disease detection [53,54]. Therefore, DTF-YOLOv11n’s favorable performance under stricter IoU conditions is consistent with the larger improvement in mAP50-95 than in mAP50.

The bounding-box loss curves of YOLOv11n and DTF-YOLOv11n were similar, whereas DTF-YOLOv11n’s classification loss decreased more markedly during the middle and later stages of training, indicating stronger category discrimination. In apple mosaic disease severity detection, phenotypic differences between adjacent severity levels are relatively subtle; therefore, enhancing informative disease-related features within the fused feature maps is particularly important for distinguishing among categories. In addition, partial convolution (PConv) in C3k2-Faster performs spatial convolution on only a subset of channels, structurally reducing redundant computation during deep feature extraction [55] and decreasing the number of model parameters by 4.63%. Because DySample and Triplet Attention add dynamic sampling and attention computations, the complete model’s FLOPs dropped by only 0.42% and remained essentially unchanged overall. Accordingly, DTF-YOLOv11n’s main improvement lies in enhancing the representation of apple mosaic disease features across different severity levels while controlling parameter scale, rather than simply reducing computational cost.

### 3.2. Performance Differences and Task Adaptability Among Different Detection Frameworks

Different detection frameworks exhibited noticeable performance differences in apple mosaic disease severity detection. RetinaNet and SSDLite achieved mAP50 values of 74.58% and 75.23%, respectively, which were lower than the 86.45–88.88% range obtained by the YOLO-series models, while DTF-YOLOv11n further increased mAP50 to 91.31%. RetinaNet achieved an R of 82.01% but a P of only 53.21%, indicating that although it maintained a relatively high target detection rate, it also generated a considerable number of incorrect predictions. SSDLite had a relatively small parameter scale, but its detection accuracy remained comparatively low. Previous studies comparing RetinaNet, SSD, and YOLO-series models for plant leaf disease detection have also reported favorable overall detection performance of the YOLO framework [56]. This is consistent with the generally higher detection accuracy observed for the YOLO-series models in this study and supports their suitability for apple mosaic disease severity detection.

Among the YOLO-series models, YOLOv11n achieved mAP50 and mAP50-95 values of 88.88% and 83.67%, respectively, both higher than the corresponding values of 86.69% and 82.23% obtained by YOLOv12n. These results indicate that updating to a newer model version does not necessarily improve apple mosaic disease severity detection performance. For this task, a model’s ability to represent fine-grained differences between adjacent severity levels is more critical than simply adopting a newer version of a general-purpose detector. In complex orchard environments, structurally optimized models tailored to specific apple leaf disease detection tasks have also achieved better detection performance than YOLOv12n, consistent with the results of this study [57]. DTF-YOLOv11n introduces targeted improvements in feature upsampling, fused feature enhancement, and deep feature extraction, thereby further improving apple mosaic disease severity detection performance. RetinaNet and SSDLite use different model architectures and training schemes; therefore, the observed performance differences reflect the overall performance of the complete detection systems on this dataset and cannot be attributed solely to a particular backbone or detection head. These framework-specific GPU timings provide an efficiency reference, but performance on agricultural edge devices remains to be evaluated.

### 3.3. Individual Effects and Combined Relationships of the Improvement Modules

The single-module ablation results showed that DySample, Triplet Attention, and FasterNet-PConv all improved apple mosaic disease severity detection performance, although their primary effects differed. Among the three single-module configurations, DySample achieved the highest mAP50 and mAP50-95 values (90.78% and 86.09%, respectively), indicating that optimizing the feature upsampling process had a relatively pronounced effect on the current task. With Triplet Attention applied after the P3/P4/P5 fused feature blocks, mAP50 and mAP50-95 reached 90.53% and 85.85%, respectively, supporting the effectiveness of this configuration for severity detection. In contrast, FasterNet-PConv mainly operated at the deep feature-extraction stage and reduced parameters to 2.458 M and FLOPs to 6.273 G, lowering model complexity while improving detection performance. Similar studies have also shown that lightweight structures, attention mechanisms, and multiscale fusion modules affect detection accuracy and model complexity differently. Lightweight structures may introduce some accuracy loss, whereas attention mechanisms and feature-fusion optimization can further improve detection performance [58].

Further combination ablation experiments showed that the performance gains of the improvement modules were not simply additive. The combination of DySample and Triplet Attention achieved mAP50 and mAP50-95 values of 91.22% and 86.15%, respectively, which were clearly higher than those obtained when either module was used alone. However, the DySample + FasterNet-PConv combination achieved mAP50 and mAP50-95 values of 90.44% and 85.94%, respectively, both lower than those of the DySample-only configuration. DySample predicts sampling offsets from its input features [53], whereas FasterNet-PConv changes upstream spatial processing through partial-channel convolution and subsequent pointwise channel mixing [55]. Because DySample predicts sampling offsets from Backbone-derived features, introducing FasterNet-PConv changes both the features passed to the detection head and the features used to determine sampling locations. Unlike diseased leaves, Healthy leaves have no mosaic or chlorosis cues; their detection relies more heavily on intact leaf appearance and separation from the orchard background. Changes in feature extraction and resampling could therefore make Healthy leaves harder to distinguish from the background. This interpretation is consistent with the test results: correctly matched Healthy leaves decreased from 131 to 122, missed Healthy leaves increased from 44 to 53, and Healthy-labelled predictions on background increased from 34 to 60. Healthy AP50 also fell from 81.37% to 74.47%, so the decline was not limited to stricter IoU thresholds. Although AP50-95 improved for Light, Moderate, and Severe, the 6.65-percentage-point decrease for Healthy outweighed those gains and lowered the four-class mAP50-95 by 0.16 percentage points. A previous apple-disease YOLOv11s study likewise reported different ablation outcomes when an attention module was used alone and in combination with a modified downsampling module [59]. The complete DTF-YOLOv11n, which integrates these established modules, achieved the highest mAP50 and mAP50-95 among the eight ablation configurations. The methodological contribution therefore lies in task-oriented network configuration and experimental validation for apple mosaic disease severity detection, while the individual modules retain their established core mechanisms. This configuration was designed to combine content-adaptive reconstruction before fusion with attention-based refinement after fusion. Restricting PConv to the Backbone’s P4/P5 stages reduces deep-stage spatial computation while retaining the original shallow feature-extraction pathway. The present ablations support the effectiveness of this configuration under the selected placements. Alternative insertion positions were not independently compared and remain a direction for further optimization.

### 3.4. Performance Differences and Error Sources Across Different Severity Categories

We observed clear differences in detection performance across severity categories. DTF-YOLOv11n achieved AP50-95 values of 94.10% and 93.27% for the Light and Moderate categories, respectively, whereas the corresponding values for the Severe and Healthy categories were 88.27% and 71.27%. Among the four categories, the Severe category showed the largest relative improvement, reaching 5.70%. These results indicate that the model improvements enhanced detection performance across different severity categories, although the detection difficulty varied among categories. Liu et al. similarly reported differences in recognition performance among five severity levels of apple frogeye leaf spot, with some levels being more easily confused with adjacent severity levels [44]. In the present dataset, the Moderate category contained 3873 annotated targets, approximately 4.87 times the 795 targets in the Severe category. Previous studies indicate that class imbalance can hinder minority-class learning in plant disease recognition [60,61], which may partly explain the lower AP50-95 of the Severe category relative to the Light and Moderate categories. However, the Healthy category contained more annotated targets than the Severe category but had lower AP50-95, indicating that sample frequency alone cannot explain the observed differences in detection performance.

Among matched leaf targets, classification errors were mainly concentrated between adjacent severity categories. After model improvement, the proportion of Severe samples classified as Moderate decreased from 0.21 to 0.18, the proportion of Light samples classified as Moderate decreased from 0.03 to 0.01, and the proportion of Moderate samples classified as Light decreased from 0.05 to 0.03. Plant disease severity generally changes continuously, and no completely distinct visual boundaries exist between adjacent severity levels. Therefore, fine-grained severity grading is more prone to confusion between adjacent levels than simple disease-category recognition.

Figure 4 provides visual examples of these remaining errors. In Figure 4a,b, the leaves were localized accurately but assigned to a lower adjacent severity category. Scattered chlorosis interspersed with green tissue in Figure 4a and uneven illumination on a curved leaf in Figure 4b may make the extent and spatial distribution of disease symptoms harder to assess. Figure 4c illustrates a different failure: a partially visible Healthy leaf near the image boundary was missed amid surrounding branches and foliage, although the Moderate leaf in the same scene was detected. Limited target visibility and background clutter may contribute to such missed detections.

The Healthy category remained relatively challenging for the current model. Although its AP50-95 increased, the diagonal proportion in the normalized confusion matrix decreased from 0.74 to 0.71, while the proportion classified as Background increased from 0.25 to 0.28. For each IoU threshold, AP integrates the interpolated precision–recall curve obtained by ranking retained detections by confidence. AP50-95 then averages AP across ten IoU thresholds from 0.50 to 0.95. In contrast, the confusion matrix counts one-to-one matches at a fixed confidence threshold of 0.25 and an IoU matching threshold of 0.45. A Healthy ground-truth leaf without a matched prediction is recorded in the Background row as a false negative, rather than an explicit background-class prediction. Although more Healthy targets were missed, unmatched predictions labelled Healthy decreased from 67 to 42. Fewer false-positive detections can improve precision over portions of the PR curve despite reduced recall under the fixed matching criteria. This precision–recall trade-off is consistent with the increased Healthy AP50-95 despite the lower diagonal proportion in the confusion matrix. Bi et al. reported that class imbalance and environmental interference, such as illumination variations and shadows, can make it harder to distinguish samples with different disease severity levels [45]. Therefore, future work should expand the number of healthy and diseased samples collected under diverse environmental conditions and use category-specific evaluation results to improve model stability across severity levels.

## 4. Materials and Methods

### 4.1. Construction of the Apple Mosaic Disease Severity Grading Dataset

**Figure 6:**
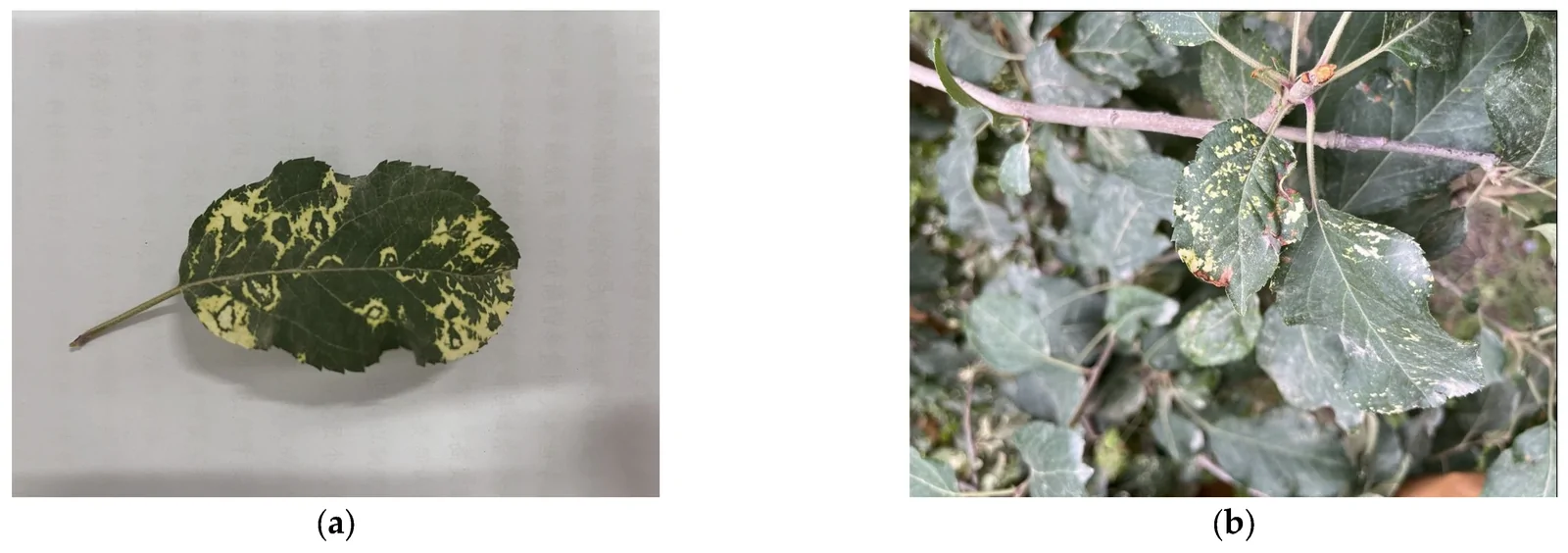
Apple leaf images acquired under different collection scenarios. (**a**) Detached leaves against a relatively uniform background. (**b**) Leaves attached to trees under natural conditions.

We collected the apple leaf images used in this study from apple orchards in Alar City, Xinjiang, China, and from Tarim University. The dataset included both images of leaves attached to trees under natural conditions and images of detached leaves acquired against a relatively uniform background, as shown in Figure 6. The natural-scene images contained overlapping branches and leaves, illumination variations, and background interference, whereas the uniform-background images provided clearer representations of leaf color, texture, and disease symptoms. Offline-augmented images were generated from the original images through horizontal flipping and noise addition. Both the original images and their offline-augmented versions were retained, resulting in a total of 6980 valid images.

Leaf disease severity was visually assessed based on the spatial extent of mosaic patterns, degree of chlorosis, necrotic symptoms, and irregular streaks along the veins [62]. We assigned leaves to four categories—Healthy, Light, Moderate, and Severe—according to the distribution and intensity of these symptoms and the extent of visibly unaffected leaf tissue. These categories represented ordinal visual symptom grades rather than classes defined by quantitatively measured lesion-to-leaf area ratios. Healthy leaves showed relatively uniform color and texture, with no obvious mosaic patterns, chlorosis, necrosis, or abnormal vein streaks. Lightly affected leaves showed scattered pale mosaic spots, mild chlorosis, or abnormal vein streaks only in localized regions. In contrast, most of the leaf surface retained normal color and texture. Moderately affected leaves showed more evident mosaic patterns, chlorosis, or streak-like symptoms in multiple regions, with a wider distribution of diseased areas and more pronounced differences in color and texture between diseased and healthy tissues. Severely affected leaves showed widespread mosaic patterns, chlorosis, or necrotic symptoms, with some diseased regions becoming interconnected and the proportion of normal leaf surface markedly reduced.

We annotated apple leaf targets with rectangular bounding boxes using LabelImg. For uniform-background images, we annotated fully visible target leaves. For natural-scene images, we annotated valid targets individually based on leaf boundaries and visual distinguishability. After annotation, the dataset contained 7968 leaf targets.

**Figure 7:**
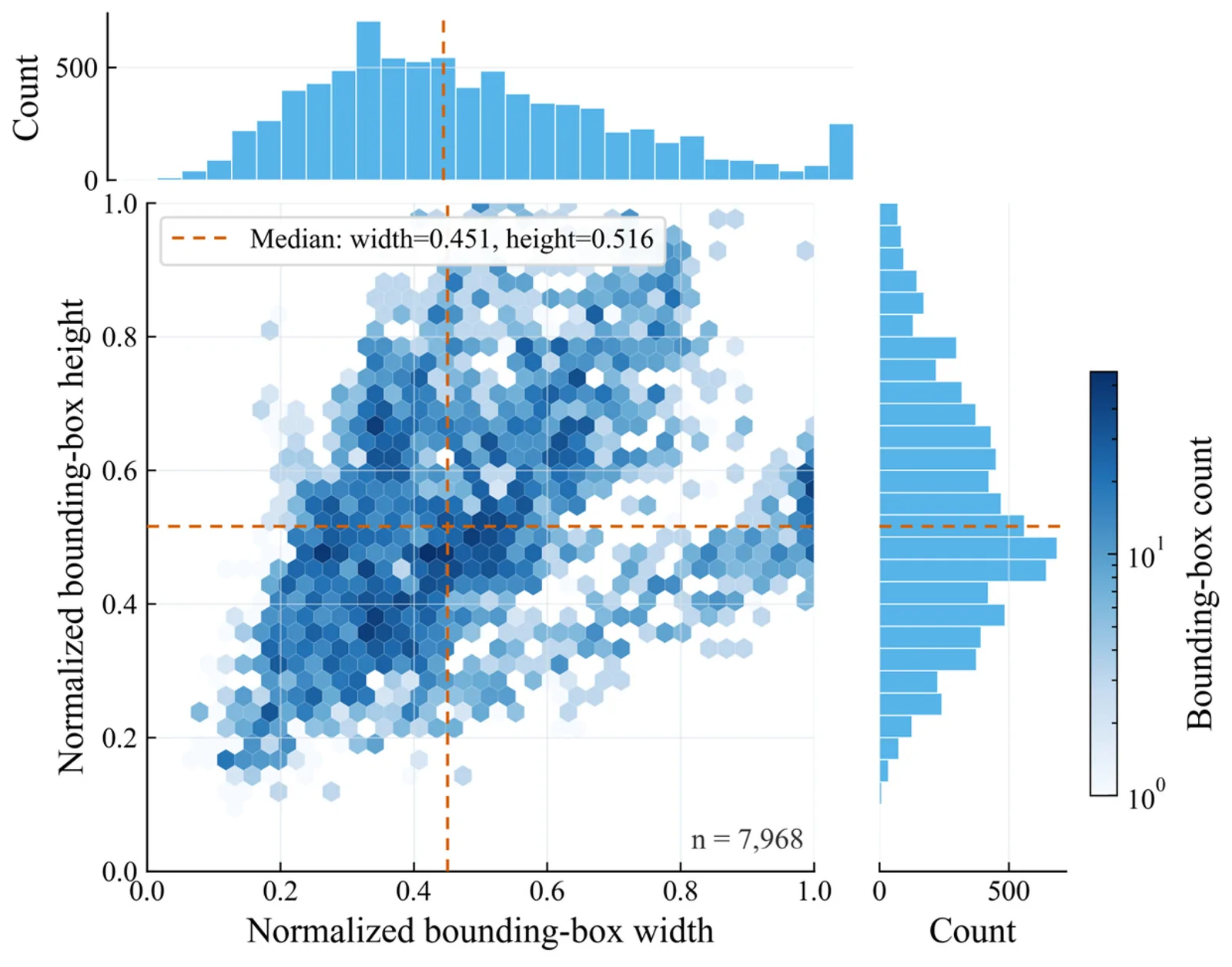
Distribution of normalized width and height of apple leaf bounding boxes.

We divided the dataset into training, validation, and test sets at a 7:1.5:1.5 ratio, containing 4886, 1047, and 1047 images, respectively. The distribution of images and annotated leaf targets across the four categories is summarized in Table 5. To prevent an original image and its augmented versions from appearing in different subsets, we grouped each original image, its horizontally flipped version, and its noise-augmented version before partitioning the dataset. We assigned all images within each group exclusively to the training, validation, or test set. Before model input, LetterBox was used for proportional resizing and border padding, standardizing the input size to 640 × 640 pixels. During training, online data augmentation, including hue–saturation–value (HSV) color augmentation, translation, scaling, horizontal flipping, and Mosaic, was applied to both original and offline-augmented images in the training subset. Mosaic was disabled during the final 10 training epochs. We applied no random augmentation to the validation or test sets. Figure 7 shows the distributions of normalized leaf target width and height. Most targets had normalized widths ranging from 0.335 to 0.610 and normalized heights ranging from 0.402 to 0.661, indicating that the dataset was dominated by medium and relatively large targets, while also containing a small number of targets that were either relatively small or occupied nearly the entire image. Differences in acquisition distance, leaf orientation, and the proportion of the image occupied by the leaf caused noticeable variations in target scale, requiring the model to support multiscale feature extraction and fusion.

### 4.2. Construction of the DTF-YOLOv11n Network

**Figure 8:**
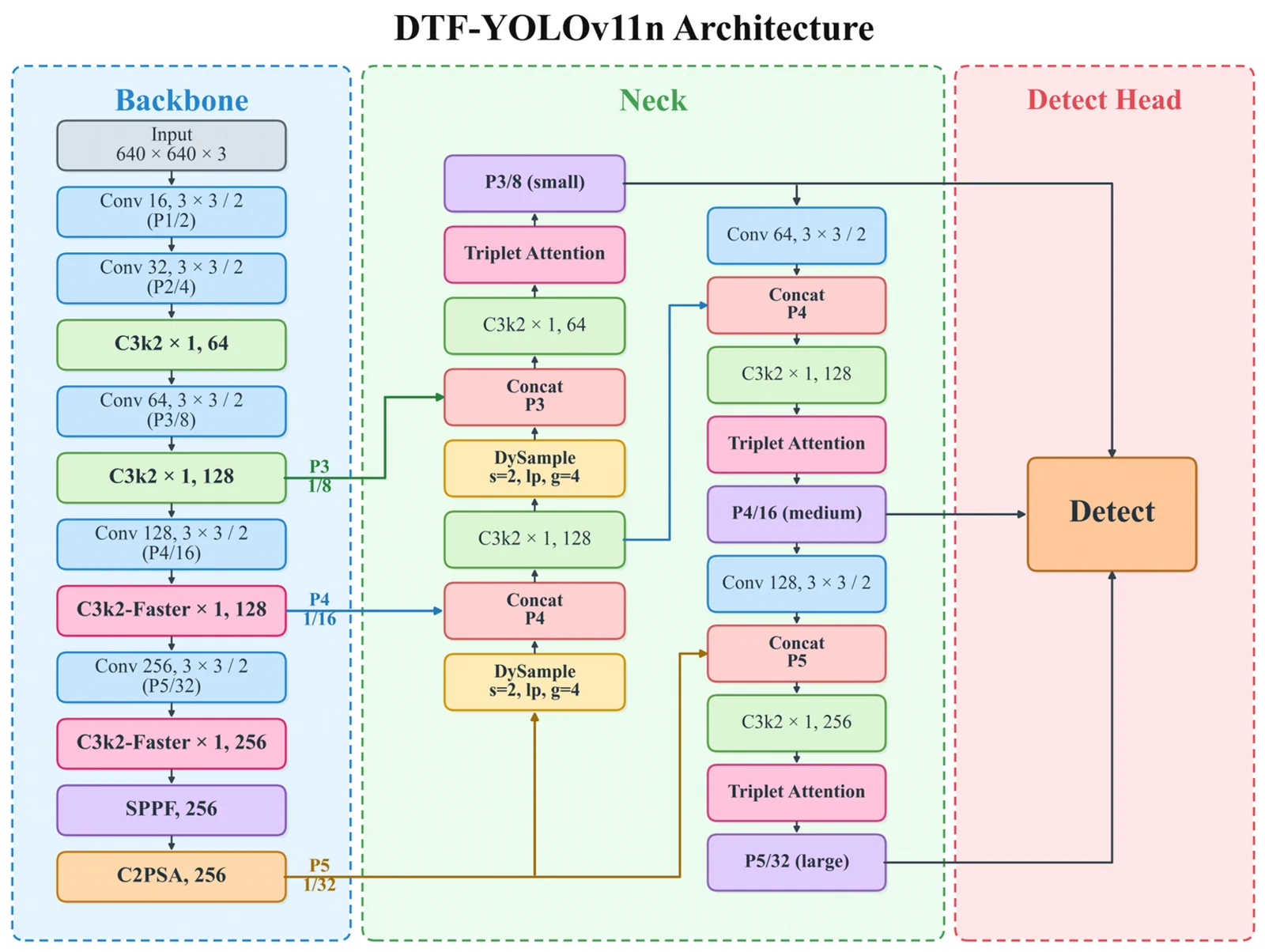
Overall architecture of DTF-YOLOv11n.

YOLOv11n mainly consists of a Backbone, Neck, and Detect Head. The Backbone extracts visual features at different levels, while the Neck fuses multiscale information through top-down and bottom-up pathways. The Detect Head performs category prediction and bounding-box regression based on the P3, P4, and P5 features [63]. Because apple mosaic disease severity detection is affected by information loss, subtle feature differences between adjacent severity levels, and channel redundancy in deep feature extraction, we developed DTF-YOLOv11n based on YOLOv11n. We introduced three structural modifications across different feature-processing stages of the Backbone and Neck. In the Neck’s top-down feature-fusion pathway, we replaced the two original fixed upsampling operations with DySample, allowing the sampling locations to adjust dynamically to the input features. We added Triplet Attention after the fused P3, P4, and P5 feature outputs to recalibrate the fused features through channel-spatial interactions. In the P4 and P5 stages of the Backbone, we replaced the original deep feature blocks with C3k2-Faster modules, using PConv to reduce spatial convolution operations. These three modifications correspond to feature upsampling, fused feature enhancement, and deep feature extraction, respectively. Figure 8 shows the overall architecture.

### 4.3. DySample Dynamic Upsampling Module

In the YOLOv11n Neck, upsampling and feature concatenation fuse features at different scales. Specifically, deep low-resolution features are upsampled and then fused with shallow high-resolution features to preserve both semantic information and spatial details. For apple mosaic disease severity grading, local chlorosis, mosaic textures, and their spatial distribution provide important cues for distinguishing adjacent severity levels [41]. However, nearest-neighbor upsampling in YOLOv11n increases feature-map resolution by replicating existing values according to fixed rules [53]. Its sampling locations do not adapt to the local symptom boundaries and texture variations that distinguish adjacent severity levels. To address this limitation, DySample improves the upsampling process from the perspective of point sampling. By learning sampling offsets in the feature space, it dynamically adjusts sampling locations based on the input content, allowing feature reconstruction to adapt to variations in input features and better preserve local details and small-scale features.

**Figure 9:**
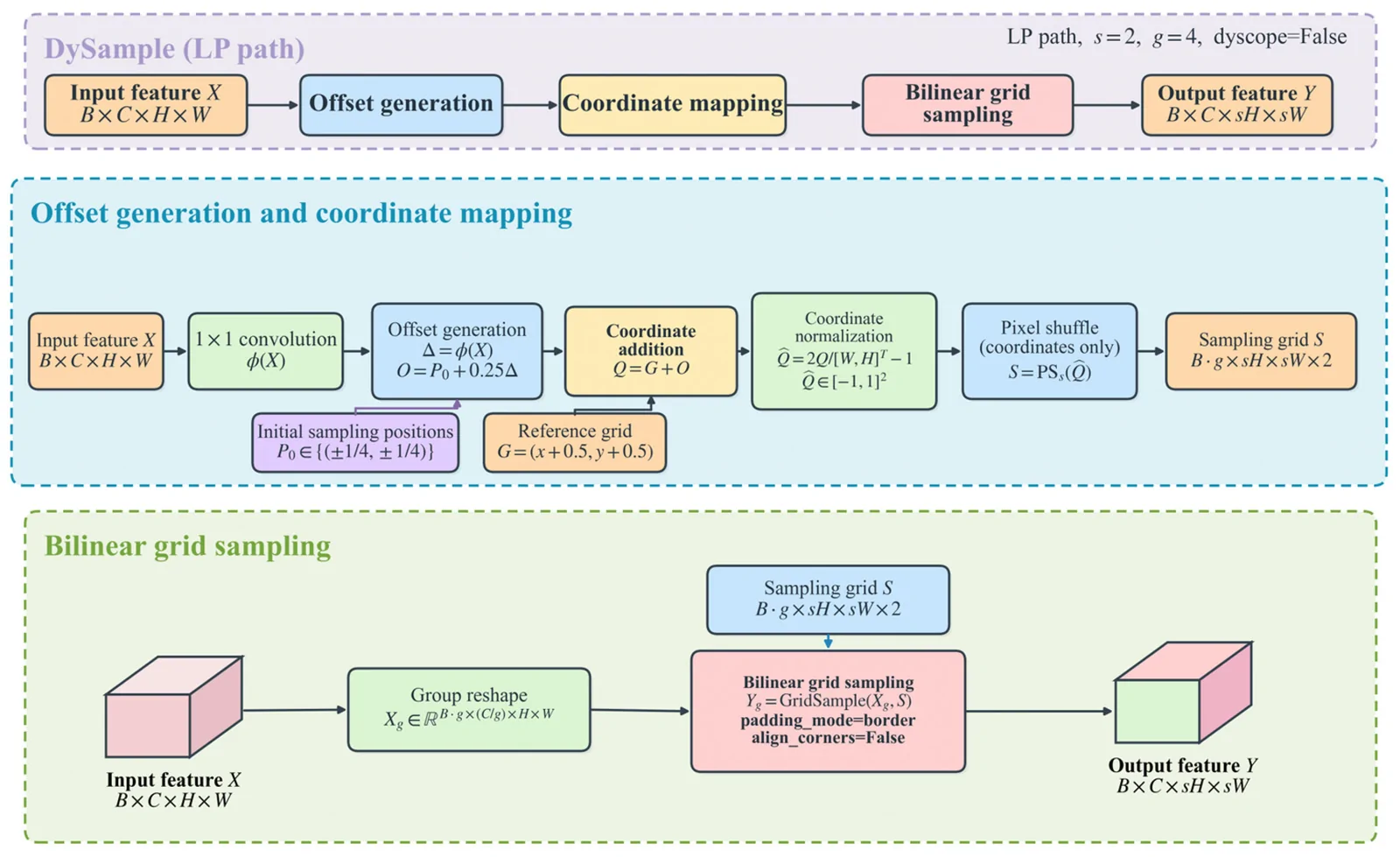
LP pathway of the DySample dynamic upsampling module.

In DTF-YOLOv11n, DySample replaces the two fixed upsampling operations in the Neck, with both modules performing 2× upsampling using the LP sampling mode and a four-group configuration. During top-down feature fusion, DySample dynamically resamples the deep semantic features, then concatenates and fuses them with the P4- and P3-scale features, respectively. Both upsampling operations were replaced to provide content-adaptive reconstruction at each resolution transition in the P5-to-P4-to-P3 top-down pathway. Similar use of DySample during feature fusion has been reported in rice leaf disease detection [51]. Replacing only one operation would reduce the additional computation but retain fixed sampling at the other transition. Figure 9 shows the structure of the DySample module.

### 4.4. Triplet Attention Mechanism

**Figure 10:**
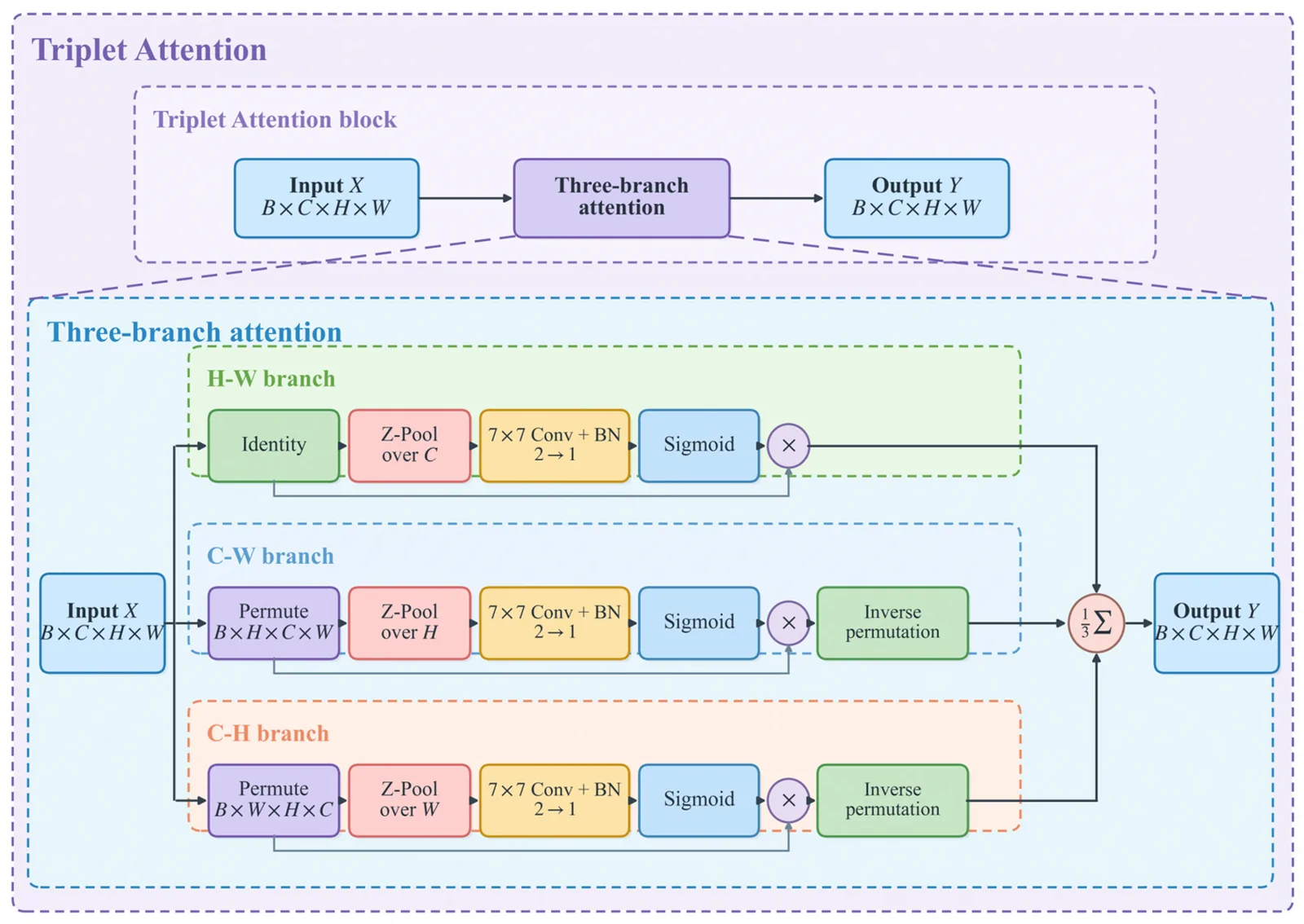
Three-branch cross-dimensional attention structure of Triplet Attention.

Phenotypic differences among severity levels of apple mosaic disease are relatively subtle, and some diseased regions are also susceptible to interference from leaf veins, illumination variations, and complex backgrounds, which increases the difficulty of distinguishing key disease-related features. To improve the network’s response to disease-relevant regions, we introduced the Triplet Attention mechanism [64]. This module consists of three parallel branches that establish cross-dimensional interactions between the channel and height, channel and width, and height and width dimensions, respectively. By integrating attention information across different dimensions, the module adaptively enhances important features while suppressing background responses unrelated to disease detection, thereby improving the network’s representation of fine-grained disease features. Figure 10 shows the structure of the Triplet Attention module.

In DTF-YOLOv11n, Triplet Attention is placed after the fused feature blocks at P3, P4, and P5 to recalibrate features at each detection scale. A similar configuration, with attention before the three detection heads, has been examined in previous YOLO-based research [65]. Here, post-fusion placement allows attention weights to be computed from features that combine spatial details and semantic information. Pre-fusion attention could filter irrelevant input responses earlier, but would not directly recalibrate the resulting fused outputs. Using attention at only one scale would reduce computational overhead but leave the remaining scales without direct attention-based refinement.

### 4.5. C3k2-Faster Lightweight Feature Extraction Module

**Figure 11:**
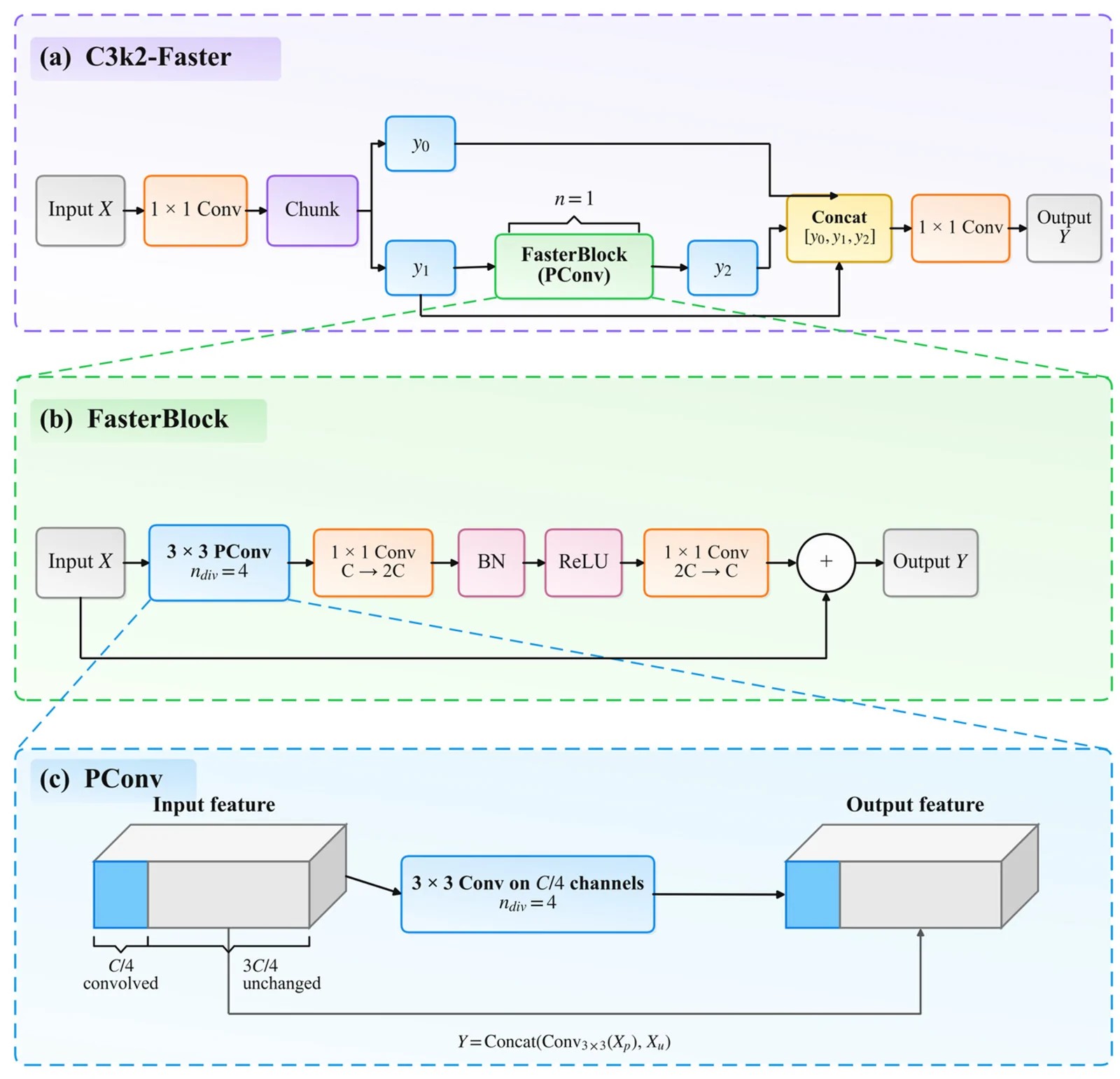
Structure of C3k2-Faster and its internal FasterBlock.

To reduce redundant computation during deep feature extraction in YOLOv11n, we constructed the C3k2-Faster module based on partial convolution (PConv) in FasterNet. We used it to replace the corresponding C3k2 modules at the P4 and P5 stages of the Backbone. The original FasterNet design allocates more blocks to its later stages based on their memory-access and computational-throughput characteristics [55]. Here, restricting C3k2-Faster to the P4 and P5 stages preserves the shallow Backbone blocks and avoids replacing the Neck feature-fusion blocks with C3k2-Faster. Applying C3k2-Faster to additional shallow Backbone or Neck blocks might reduce spatial-convolution operations, but would also alter high-resolution feature extraction or multiscale fusion; the effects of these alternative placements on accuracy and efficiency were not experimentally evaluated in this study. C3k2-Faster retains the original branch and feature concatenation structure of C3k2, while introducing FasterBlock only into the feature transformation branch. Given the relatively large number of channels at the P4 and P5 stages, applying spatial convolution to all channels would incur considerable computational overhead. Therefore, only a subset of channels undergoes convolution. Figure 11 shows the structure. After splitting the input feature along the channel dimension, only part of the channels undergo 3 × 3 convolution, while the remaining channels are preserved directly; the two parts are then concatenated. In this study, we set n_div to 4, meaning one-quarter of the channels participated in spatial convolution. The concatenated features were then processed by a pointwise multilayer perceptron (MLP) with an expansion ratio of 2. Its structure comprised a 1 × 1 expansion convolution, batch normalization (BN), a rectified linear unit (ReLU), and a second 1 × 1 convolution restoring the original channel dimension. The MLP output was added to the FasterBlock input through a residual connection. By reducing the number of channels in spatial convolution, this structure reduces parameters and computational overhead during deep feature extraction while avoiding substantial changes to the original feature propagation pathway.

### 4.6. Experimental Setup

All experiments were conducted on Windows 11 using an Intel Core i5-12400F processor and an NVIDIA GeForce RTX 4060 8 GB GPU. The software environment consisted of Python 3.10.20 and PyTorch 2.5.1. YOLOv5nu, YOLOv8n, YOLOv10n, YOLOv11n, and DTF-YOLOv11n were implemented using Ultralytics 8.3.33. We implemented YOLOv12n using its officially released code, which is based on Ultralytics 8.3.63. All YOLO-series models used their corresponding official pretrained weights. We set the input image size to 640 × 640 pixels; trained the models for 100 epochs with a batch size of 16; set the number of data-loading workers to 8; and enabled automatic mixed-precision training. We used AdamW as the optimizer, with an initial learning rate of 0.00125, a first-moment decay coefficient (β1) of 0.9, a weight decay of 0.0005, and 3 warm-up epochs. Aside from differences in model architecture and specific code implementation, all YOLO-series models used the same dataset split and key training settings.

The comparison models included YOLOv5nu, YOLOv8n, YOLOv10n, YOLOv11n, YOLOv12n, RetinaNet, and SSDLite. RetinaNet is a one-stage object detection model based on a ResNet-50 backbone and a feature pyramid network, performing object classification and bounding-box regression using multiscale features [66]. SSDLite uses MobileNetV3-Large as its backbone and employs lightweight convolutional structures for multiscale object detection [67]. Both non-YOLO models were implemented using torchvision and employed their respective pretrained weights, model input transformations, and loss formulations. All models used the same dataset split and category definitions and were evaluated on the same test set using consistent evaluation metrics.

Inference speed was obtained from the saved test-set evaluation records using a batch size of 1 on the same GPU. GPU latency was also extracted from these records and reported as the average processing time per image under the corresponding timing scope. For YOLO-series models, FPS was calculated from the framework-reported inference time, excluding separately timed preprocessing and postprocessing. For RetinaNet and SSDLite, timing covered the native detector call, including internal input transformations and postprocessing, with GPU synchronization and the first batch excluded. These timing definitions are distinguished in Table 2, and neither FPS nor latency values are directly compared across different timing scopes.

### 4.7. Model Evaluation Metrics

To evaluate model performance in apple mosaic disease severity detection, we used P, R, mAP50, and mAP50-95. P represents the proportion of correctly detected targets among all predicted targets, whereas R represents the proportion of correctly detected targets among all ground-truth targets. AP is calculated as the area under the PR curve. mAP50 represents the mean AP across all categories at an IoU threshold of 0.50, while mAP50-95 represents the mean AP across all categories at IoU thresholds ranging from 0.50 to 0.95 with a step size of 0.05. The confusion matrices for YOLOv11n and DTF-YOLOv11n used a confidence threshold of 0.25 and an IoU matching threshold of 0.45, with column-wise normalization by ground-truth category. The corresponding formulas are as follows:

(1) $$P=\frac{\mathrm{TP}}{\mathrm{TP}+\mathrm{FP}}$$

(2) $$R=\frac{\mathrm{TP}}{\mathrm{TP}+\mathrm{FN}}$$

(3) $$\mathrm{mAP}50=\frac{1}{n}\sum_{i=1}^{n}AP_{i}$$

(4) $$\mathrm{mAP}50\text{–}95=\frac{1}{n}\sum_{i=1}^{n}\frac{1}{10}\sum_{j=1}^{10}AP_{i,j}$$

where *TP* (true positives), *FP* (false positives), and *FN* (false negatives) denote the numbers of correctly detected, incorrectly detected, and missed targets, respectively; n is the number of categories; *AP_i_* denotes the *AP* of the i-th category at an IoU threshold of 0.50; *AP_i,j_* denotes the *AP* of the i-th category at the j-th IoU threshold; and mAP50-95 represents the mean AP obtained across IoU thresholds ranging from 0.50 to 0.95 with a step size of 0.05.

## 5. Conclusions

To address subtle phenotypic differences across apple mosaic disease severity levels and the attenuation of local disease information during feature propagation and upsampling, DTF-YOLOv11n was developed by integrating DySample, Triplet Attention, and FasterNet-PConv into YOLOv11n. The network was optimized at three stages: feature upsampling, fused feature enhancement, and deep feature extraction. The experimental results showed that DTF-YOLOv11n achieved mAP50 and mAP50-95 values of 91.31% and 86.73%, respectively, representing relative improvements of 2.73% and 3.66% over YOLOv11n. The number of parameters decreased from 2.591 M to 2.471 M, corresponding to a reduction of 4.63%, while FLOPs remained essentially unchanged. The ablation experiments showed that all three structural modifications improved detection performance, and the complete model achieved the highest mAP50 and mAP50-95 among all ablation configurations.

The category-specific results showed that DTF-YOLOv11n improved AP50-95 for the Healthy, Light, Moderate, and Severe categories compared with the baseline model, with the Severe category showing the largest relative improvement. Compared with YOLOv5nu, YOLOv8n, YOLOv10n, YOLOv11n, YOLOv12n, RetinaNet, and SSDLite, DTF-YOLOv11n achieved the highest mAP50 and mAP50-95. Overall, the proposed model improved detection accuracy across apple mosaic disease severity levels while controlling parameter scale, demonstrating its potential for automated severity detection and disease monitoring.

This study still has several limitations. Current severity determination relies primarily on visible phenotypic information in red–green–blue (RGB) images, which makes it difficult to detect early or latent infections before obvious symptoms appear and does not capture viral load or internal physiological changes in leaves. In addition, the current model is supervised with discrete severity labels and can output only four categories—Healthy, Light, Moderate, and Severe—rather than directly estimating continuous severity values or the proportion of diseased area. Because these grades were visually defined without quantitative area thresholds, the boundaries between adjacent grades may be affected by observer judgment. Future research could integrate lesion segmentation, continuous severity estimation, and spectral information to support more quantitative severity assessment. Although the dataset includes both natural orchard images and detached-leaf images acquired against a relatively uniform background, broader applicability requires independent validation across orchards, acquisition periods, illumination conditions, and apple cultivars. Future evaluations should use data from orchards excluded from model development and cover different seasons and shooting angles to assess the effects of variations in symptom visibility, leaf color, and texture on grading consistency and field robustness.

## Figures and Tables

**Table 1:** Comparison of detection performance between YOLOv11n and DTF-YOLOv11n.

Model	P/%	R/%	mAP50/%	mAP50-95/%	Params/M	FLOPs/G	Model Size/MB
YOLOv11n	86.70	85.71	88.88	83.67	2.591	6.444	5.238
DTF-YOLOv11n	91.40	86.41	91.31	86.73	2.471	6.417	5.010

**Table 2:** Performance comparison of different object detection models.

Model	P/%	R/%	mAP50/%	mAP50-95/%	Params/M	FLOPs/G	Model Size/MB	FPS	GPU Latency (ms/Image)
RetinaNet	53.21	82.01	74.58	64.96	36.415	—	139.237	19.0 ^b^	52.57 ^b^
SSDLite	52.26	86.13	75.23	57.16	2.248	—	8.868	67.2 ^b^	14.88 ^b^
YOLOv5nu	88.37	79.76	86.45	81.31	2.509	7.180	5.042	153.8 ^a^	6.50 ^a^
YOLOv8n	86.94	83.67	87.12	82.18	3.012	8.197	5.972	158.3 ^a^	6.32 ^a^
YOLOv10n	86.70	83.17	87.67	82.09	2.709	8.399	5.511	107.9 ^a^	9.27 ^a^
YOLOv11n	86.70	85.71	88.88	83.67	2.591	6.444	5.238	140.8 ^a^	7.10 ^a^
YOLOv12n	84.83	84.29	86.69	82.23	2.520	5.982	5.218	85.9 ^a^	11.64 ^a^
DTF-YOLOv11n	91.40	86.41	91.31	86.73	2.471	6.417	5.010	106.2 ^a^	9.42 ^a^

Notes: ^a^ Framework-reported inference-stage timing, excluding separately timed preprocessing and postprocessing. ^b^ Native detector-call timing, including internal input transformations and postprocessing. FPS and GPU latency were obtained from saved test-set evaluation records; values with different timing scopes are not directly comparable. — indicates unavailable FLOP values.

**Table 3:** Results of the DTF-YOLOv11n ablation experiments.

DySample	Triplet Attention	FasterNet-PConv	P/%	R/%	mAP50/%	mAP50-95/%	Params/M	FLOPs/G
—	—	—	86.70	85.71	88.88	83.67	2.591	6.444
√			91.95	85.59	90.78	86.09	2.603	6.461
	√		88.46	87.10	90.53	85.85	2.592	6.571
		√	89.92	84.24	90.22	85.38	2.458	6.273
√	√		92.20	84.63	91.22	86.15	2.604	6.588
√		√	91.60	85.62	90.44	85.94	2.470	6.290
	√	√	91.65	85.99	90.74	86.02	2.459	6.400
√	√	√	91.40	86.41	91.31	86.73	2.471	6.417

**Table 4:** Detection performance for different apple mosaic disease severity levels.

Severity Level	YOLOv11n AP50-95/%	DTF-YOLOv11n AP50-95/%	Relative Improvement/%
Healthy	69.55	71.27	+2.47
Light	90.68	94.10	+3.77
Moderate	90.93	93.27	+2.57
Severe	83.51	88.27	+5.70

**Table 5:** Distribution of images and annotated leaf targets across severity categories.

Severity Category	Images Containing the Category	Annotated Leaf Targets
Healthy	1109	1164
Light	2099	2136
Moderate	3779	3873
Severe	784	795

## Data Availability

The data presented in this study are available on request from the corresponding author.
